# Digital health technology in clinical trials

**DOI:** 10.1038/s41746-023-00841-8

**Published:** 2023-05-18

**Authors:** Mirja Mittermaier, Kaushik P. Venkatesh, Joseph C. Kvedar

**Affiliations:** 1 Department of Infectious Diseases, Respiratory Medicine and Critical Care, Charité - Universitätsmedizin Berlin, Corporate Member of Freie Universität Berlin and Humboldt-Universität zu Berlin, Berlin, Germany; 2 Berlin Institute of Health at Charité – Universitätsmedizin Berlin, Charitéplatz 1, 10117 Berlin, Germany; 3 Harvard Medical School, Boston, MA USA

**Keywords:** Diagnostic markers, Clinical trials, Diagnostic markers

## Abstract

Digital health technologies (DHTs) have brought several significant improvements to clinical trials, enabling real-world data collection outside of the traditional clinical context and more patient-centered approaches. DHTs, such as wearables, allow the collection of unique personal data at home over a long period. But DHTs also bring challenges, such as digital endpoint harmonization and disadvantaging populations already experiencing the digital divide. A recent study explored the growth trends and implications of established and novel DHTs in neurology trials over the past decade. Here, we discuss the benefits and future challenges of DHT usage in clinical trials.

Digital health technologies (DHTs) have enabled a cornucopia of new data opportunities for clinical trials. DHTs range widely, including software (e.g., mobile health apps), hardware (e.g., wearable devices, sensors), and telemedicine platform solutions. In neurology trials, DHTs have already been shown to provide better data from “real-life” settings^1^. Advances in DHTs have started to percolate through clinical trial design and enable more patient-centered research^2^ and real-world data-driven decisions. As more clinical trials adopt DHTs, understanding their benefits and challenges is valuable for patients, physicians, and clinical researchers^3^. Masanneck et al.^3^ recently analyzed the evolution of DHTs utilized in neurology trials over the last decade.

## Evolution of DHT usage in clinical trials

Traditionally, data are collected in clinical visits capturing only a single time point or limited timeframe. These clinical visits present logistical and financial barriers for subjects, particularly those with high morbidity. DHTs allow continuous remote monitoring of patients’ health data while they continue their daily lives. These novel measurements can provide insights into disease physiology and outcomes. Indeed, this new era of DHT-generated data can allow for digital phenotyping, i.e., the quantification of individual patients using multimodal data from personal digital devices^4^, and can help build digital twins for precision medicine^5,6^.

Masanneck et al.^3^ analyzed the evolution of the use of DHTs in trials registered on ClinicalTrials.gov for four chronic neurological disorders: epilepsy, multiple sclerosis, Alzheimer’s disease, and Parkinson’s disease. They found that the relative frequency of clinical trials using DHTs increased from 0.7% in 2010 to 11.4% in 2020. Kaiser Associates projected that up to 70% of clinical trials will incorporate wearable sensors by 2025^7^. Masanneck et al.^3^ further described a trend from simple tracking methods such as motor function and exercise patterns in 2010 towards more complex methods like speech and cognition tracking. Together, the authors showed the growth of DHTs in neurology trials and an increase in disease-specific digital measurements.

## Access and barriers to decentralized and virtual clinical trials

DHTs enable clinical trials to occur anywhere at any time^8^. For patients, decentralized and virtual trial settings can reduce the burden of trial participation by, e.g., reducing time and costs spent to travel^9^, and accelerating the pace of clinical research^10^.

On the other hand, trials using DHTs might also disadvantage groups who have limited access to the internet or sparse technology literacy^11,12^. *The Lancet* and *Financial Times* Commission report on “Governing health futures 2030: Growing up in a digital world” recommends investing in the enablers of a digital transformation of public health and Universal Health Coverage in line with country roadmaps, working towards a robust national digital infrastructure. Disconnection from online services adds up to the digital divide. Infrastructure providing reliable and affordable internet in highly vulnerable areas would be a key step in bridging this divide.

## Novel digital endpoints and regulatory approaches

DHTs present the opportunity to use novel clinical trial endpoints to generate real-world evidence. A critical part of clinical trials is thoughtful study design, including primary and secondary endpoints that are reliable and reflective of the study objectives. Additionally, standardized terminology and best practices for the evaluation of Biometric Monitoring Technologies (BioMeTs)—a subgroup of DHTs—are necessary to build trust and comparability across communities. A three-component framework (V3), including (1) verification, (2) analytical validation, and (3) clinical validation steps, can be used to develop evaluation methodologies for the clinical and scientific utility of BioMeTs^13^. Moreover, a standardized evaluation framework necessary for algorithms developed and used in the context of BioMeTs^14^ - trustworthiness, explainability, usability, and transparency should be addressed^15^.

Further, adapted regulatory guidelines are necessary to clarify and simplify the market entry of DHTs in validating trials. A significant step in this arena was the U.S. Food and Drug Administration (FDA) 2021 draft guidance on “Digital Health Technologies for Remote Data Acquisition in Clinical Investigations”^16^. This new guidance provides recommendations ranging from endpoint collection with DHTs to verification, validation, and usability of DHTs in clinical trials.

## Participant authentication and data reliability

Remote data collection in virtual clinical trials raises specific challenges in terms of authentication. Wearables could be used by users different from the designated subject, a significant threat to the validity of trial data collection. Biometric authentication such as fingerprint or iris scanners have been already used to positively identify patients at on-site clinical trials^17,18^. These technologies have yet to be widely used in virtual clinical trials and could be a key innovation. Recent studies successfully demonstrated that continuously acquired data itself could be used for continuous authentication. For example, AI models can identify specific users using biometric data on ambulation and heart beat acquired via wrist-worn wearable^19,20^. Although this technology is available, privacy and usability aspects require attention before their wide application in clinical trials^21^.

In contrast to data collection in the traditional laboratory settings, data quality remains one of the most challenging factors that impacts data reliability in real-world settings. Artifacts can be derived from the environment (e.g., increased temperature sensing when the device is worn under a blanket), the device itself, or from the patient (e.g., improperly worn devices)^22^. Further, the lack of data completeness can also significantly impact data quality, and thus data completeness should be calculated^22^. A user might not wear a wrist sensor at night as the sensor needs to be charged or during water activities like showering and swimming if the sensor is not water resistant. A recent study evaluated the data quality from wrist-worn non-EEG wearable devices used for seizure monitoring in epilepsy patients^22^. They developed a methodology for qualitative visualization and quantitative analysis of wearable artifacts to generate a signal quality index that could be used to compare study results.

## Conclusion

In conclusion, DHT usage in clinical trials has increased over the last decade and continues to grow and evolve. DHTs enable investigators to collect continuously heterogeneous data in real-world settings, allowing the acquisition of data types previously impossible. Further, DHTs have been shown to accelerate patient recruitment. DHT-derived measures can also improve existing endpoints and develop new ones. Importantly, DHTs should be equitably deployed in trials to bridge rather than deepen the digital divide, which may require significant social investment from trial sponsors with support and guidance from government. DHTs usage in clinical trials have the potential to transform clinical trials and usher in the virtual era of distributed clinical trials.

## References

[1] Dodge HH (2015). Use of high-frequency in-home monitoring data may reduce sample sizes needed in clinical trials. PLoS ONE.

[2] Stroud C, Onnela JP, Manji H (2019). Harnessing digital technology to predict, diagnose, monitor, and develop treatments for brain disorders. NPJ Digit. Med..

[3] Masanneck L, Gieseler P, Gordon WJ, Meuth SG, Stern AD (2023). Evidence from ClinicalTrials.gov on the growth of digital health technologies in neurology trials. NPJ Digit. Med..

[4] Torous J, Kiang MV, Lorme J, Onnela JP (2016). New tools for new research in psychiatry: a scalable and customizable platform to empower data driven smartphone research. JMIR Ment. Health.

[5] Venkatesh KP, Raza MM, Kvedar JC (2022). Health digital twins as tools for precision medicine: Considerations for computation, implementation, and regulation. NPJ Digit. Med..

[6] Adamo JE (2020). Translation of digital health technologies to advance precision medicine: informing regulatory science. Digit. Biomark..

[7] Chandrasekaran R, Katthula V, Moustakas E (2020). Patterns of use and key predictors for the use of wearable health care devices by US adults: insights from a national survey. J. Med. Internet Res..

[8] De Brouwer W, Patel CJ, Manrai AK, Rodriguez-Chavez IR, Shah NR (2021). Empowering clinical research in a decentralized world. NPJ Digit. Med..

[9] Goodson N (2022). Opportunities and counterintuitive challenges for decentralized clinical trials to broaden participant inclusion. NPJ Digit. Med..

[10] Marra C, Chen JL, Coravos A, Stern AD (2020). Quantifying the use of connected digital products in clinical research. NPJ Digit. Med..

[11] Reiners F, Sturm J, Bouw LJW, Wouters EJM (2019). Sociodemographic factors influencing the use of ehealth in people with chronic diseases. Int. J. Environ. Res. Public Health.

[12] Saeed SA, Masters RM (2021). Disparities in health care and the digital divide. Curr. Psychiatry Rep..

[13] Goldsack JC (2020). Verification, analytical validation, and clinical validation (V3): the foundation of determining fit-for-purpose for Biometric Monitoring Technologies (BioMeTs). NPJ Digit. Med..

[14] Godfrey A (2020). BioMeT and algorithm challenges: a proposed digital standardized evaluation framework. IEEE J. Transl. Eng. Health Med..

[15] Cutillo CM (2020). Machine intelligence in healthcare-perspectives on trustworthiness, explainability, usability, and transparency. NPJ Digit. Med..

[16] FDA. Digital health technologies for remote data acquisition in clinical investigations. www.fda.gov/media/155022/download (2021).

[17] SonLa Study G (2007). Using a fingerprint recognition system in a vaccine trial to avoid misclassification. Bull. World Health Organ..

[18] Zola Matuvanga T (2021). Use of iris scanning for biometric recognition of healthy adults participating in an Ebola vaccine trial in the Democratic Republic of the Congo: mixed methods study. J. Med. Internet Res..

[19] Zhao, T. et al. TrueHeart: continuous authentication on wrist-worn wearables using PPG-based biometrics. *IEEE Xplore*10.1109/INFOCOM41043.2020.9155526 (2020).

[20] Zeng, Y., Pande, A., Zhu, J. & Mohapatra, P. WearIA: wearable device implicit authentication based on activity information. *IEEE Xplore*10.1109/WoWMoM.2017.7974305 (2017).

[21] Baig AF, Eskeland S (2021). Security, privacy, and usability in continuous authentication: a survey. Sensors.

[22] Bottcher S (2022). Data quality evaluation in wearable monitoring. Sci. Rep..

